# *N*,*N*′-Bis(3,4-dimethoxy­benzyl­idene)butane-1,4-diamine

**DOI:** 10.1107/S1600536809025069

**Published:** 2009-07-04

**Authors:** Aliakbar Dehno Khalaji, Karla Fejfarova, Michal Dusek

**Affiliations:** a Department of Chemistry, Faculty of Science, Golestan University, Gorgan, Iran; b Institute of Physics of the ASCR, Na Slovance 2, 182 21 Praha 8, Czech Republic

## Abstract

The title Schiff base compound, C_22_H_28_N_2_O_4_, was synthesized by the reaction of 3,4-dimethoxy­benzaldehyde and 1,4-diamino­butane in methanol. The mol­ecule is located on a center of inversion with one half-mol­ecule in the asymmetric unit. Both C=N double bonds are in a *trans* configuration. Inter­molecular C—H⋯O hydrogen bonds link the mol­ecules into a three-dimensional network.

## Related literature

For related structures, see: Khalaji & Ng (2008 ▶); Khalaji *et al.* (2007 ▶).
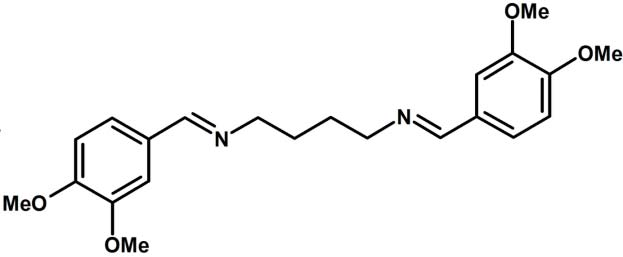


## Experimental

### 

#### Crystal data

C_22_H_28_N_2_O_4_
*M* *_r_* = 384.5
Monoclinic, 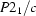

*a* = 14.5770 (4) Å
*b* = 7.6201 (2) Å
*c* = 9.4456 (3) Å
β = 101.725 (2)°
*V* = 1027.31 (5) Å^3^
*Z* = 2
Cu *K*α radiation
μ = 0.69 mm^−1^
*T* = 120 K
0.29 × 0.16 × 0.09 mm

#### Data collection

Oxford Diffraction Gemini diffractometer with Xcalibur goniometer, an Atlas detector and a Gemini ultra Cu source
Absorption correction: multi-scan (*CrysAlis RED*; Oxford Diffraction, 2009 ▶) *T*_min_ = 0.765, *T*_max_ = 0.935
5312 measured reflections
1594 independent reflections
1293 reflections with *I* > 3σ(*I*)
*R*_int_ = 0.024
θ_max_ = 62.3°

#### Refinement

*R*[*F* ^2^ > 2σ(*F* ^2^)] = 0.035
*wR*(*F* ^2^) = 0.096
*S* = 1.67
1594 reflections
127 parameters
H-atom parameters constrained
Δρ_max_ = 0.13 e Å^−3^
Δρ_min_ = −0.14 e Å^−3^

### 

Data collection: *CrysAlis CCD* (Oxford Diffraction, 2009 ▶); cell refinement: *CrysAlis RED* (Oxford Diffraction, 2009 ▶); data reduction: *CrysAlis RED*; program(s) used to solve structure: *SIR2002* (Burla *et al.*, 2003 ▶); program(s) used to refine structure: *JANA2006* (Petříček *et al.*, 2006 ▶); molecular graphics: *DIAMOND* (Brandenburg & Putz, 2005 ▶); software used to prepare material for publication: *JANA2006*.

## Supplementary Material

Crystal structure: contains datablocks global, I. DOI: 10.1107/S1600536809025069/bt2985sup1.cif

Structure factors: contains datablocks I. DOI: 10.1107/S1600536809025069/bt2985Isup2.hkl

Additional supplementary materials:  crystallographic information; 3D view; checkCIF report

## Figures and Tables

**Table 1:** Hydrogen-bond geometry (Å, °)

*D*—H⋯*A*	*D*—H	H⋯*A*	*D*⋯*A*	*D*—H⋯*A*
C11—H11A⋯O1^i^	0.96	2.54	3.4945 (17)	171
C11—H11B⋯O1^ii^	0.96	2.58	3.4830 (17)	158

Symmetry codes: (i) 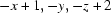; (ii) 
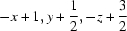
.

**Fig. 1.**
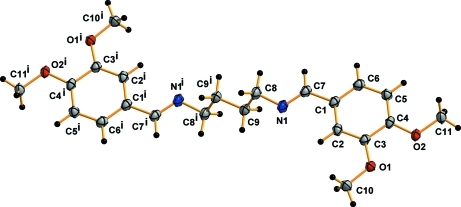
The molecular structure of the title compound with atom-labeling scheme. Displacement ellipsoids are drawn at the 50% probability level. [Symmetry codes: (i) -x, -y, -x]

**Fig. 2.**
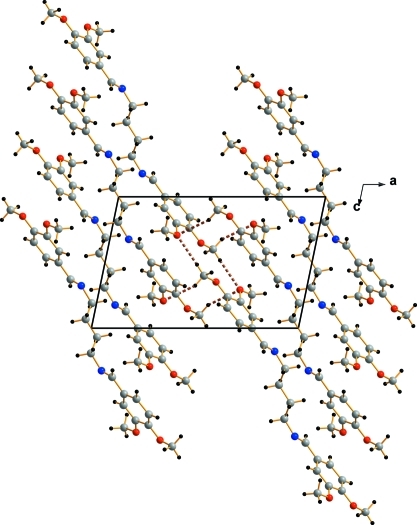
The packing of (I) viewed along b, with hydrogen bonds shown as dashed lines.

## supplementary crystallographic information

### Comment

Recently, we have synthesized Schiff base compounds with a 1,2-diaminoethane unit and analyzed their crystal structures (Khalaji & Ng, 2008; Khalaji *et al.*, 2007). In continuation of these studies, the title compound was prepared and its structure was determined.

The molecule of the title compound is shown in Fig.1. A l l bond lengths and angles are comparable with those observed in similar compounds (Khalaji & Ng, 2008; Khalaji *et al.*, 2007). The C7—N1 and C8—N1 bond lengths of 1.2657 (19) and 1.4620 (17) Å, respectively, conform to the value for a double and single bond. The molecule displays an *E *configuration about the C=N double bond.

Oxygen O1 and methyl C11H_3_ participate in two symmetry independent hydrogen bonds C—H···O which connect the molecules into a three-dimensional network (Fig. 2).

### Experimental

A solution of 3,4-dimethoxybenzaldehyde (3.30 g, 0.02 mol) in 40 ml me thanol was heated for 25 min at 65°C and then stirred for about 30 min. To this stirring solution, a solution of 1,4-diaminobutane (0.84 g, 0.01 mol) in 5 ml me thanol was added dropwise with constant stirring. The mixture was refluxed for 1 h and then allowed to cool for overnight at 298 K. The resulting crude solid was collected by filtration and dried at room temperature. Crystals were grown by the slow evaporation technique at room temperature by using a mixture of 40 ml of chloroform-methanole (5:3 *v*/*v*) as a solvent. At the period of super saturation, tiny crystals were nucleated. They were allowed to grow to a maximum possible dimension and then filtered. Yield: 3.28 g, 85%. 1*H*-NMR (CDCl3, δ(p.p.m.)): 1.75 (t, 2H8), 3.61 (t, 4H1), 3.87 (s, 6H6), 3.90 (s, 6H7), 6.84 (d, 2H3), 7.10 (dd, 2H4), 7.39 (d, 2H5), 8.16 (S, 2H2).

### Refinement

All hydrogen atoms were discernible in difference Fourier maps and could be refined to reasonable geometry. According to common practice they were nevertheless kept in ideal positions during the refinement. The isotropic atomic displacement parameters of hydrogen atoms were set to 1.2*U*_eq_ of the parent atom.

### Crystal data

**Table:**

C_22_H_28_N_2_O_4_	*F*(000) = 412
*M**_r_* = 384.5	*D*_x_ = 1.243 Mg m^−^^3^
Monoclinic, *P*2_1_/*c*	Cu *K*α radiation, λ = 1.54184 Å
Hall symbol: -P 2ybc	Cell parameters from 3320 reflections
*a* = 14.5770 (4) Å	θ = 3.1–62.2°
*b* = 7.6201 (2) Å	µ = 0.69 mm^−^^1^
*c* = 9.4456 (3) Å	*T* = 120 K
β = 101.725 (2)°	Prism, colorless
*V* = 1027.31 (5) Å^3^	0.28 × 0.16 × 0.09 mm
*Z* = 2	

### Data collection

**Table:**

Oxford Diffraction Gemini diffractometer with Xcalibur goniometer, an Atlas detector and a Gemini ultra Cu source	1594 independent reflections
Radiation source: X-ray tube	1293 reflections with *I* > 3σ(*I*)
mirror	*R*_int_ = 0.024
Detector resolution: 20.7567 pixels mm^-1^	θ_max_ = 62.3°, θ_min_ = 6.2°
Rotation method data acquisition using ω scans	*h* = −15→16
Absorption correction: multi-scan (*CrysAlis RED*; Oxford Diffraction, 2009)	*k* = −6→8
*T*_min_ = 0.765, *T*_max_ = 0.935	*l* = −10→8
5312 measured reflections	

### Refinement

**Table:**

Refinement on *F*^2^	56 constraints
*R*[*F*^2^ > 2σ(*F*^2^)] = 0.035	H-atom parameters constrained
*wR*(*F*^2^) = 0.096	Weighting scheme based on measured s.u.'s *w* = 1/[σ^2^(*I*) + 0.0016*I*^2^]
*S* = 1.67	(Δ/σ)_max_ = 0.010
1594 reflections	Δρ_max_ = 0.13 e Å^−^^3^
127 parameters	Δρ_min_ = −0.14 e Å^−^^3^
0 restraints	

### Special details

**Table:**

Refinement. The refinement was carried out against all reflections. The conventional *R*-factor is always based on *F*. The goodness of fit as well as the weighted *R*-factor are based on *F* and *F*^2^ for refinement carried out on *F* and *F*^2^, respectively. The threshold expression is used only for calculating *R*-factors *etc*. and it is not relevant to the choice of reflections for refinement.The program used for refinement, Jana2006, uses the weighting scheme based on the experimental expectations, see _refine_ls_weighting_details, that does not force *S* to be one. Therefore the values of *S* are usually larger than the ones from the *SHELX* program.

### Fractional atomic coordinates and isotropic or equivalent isotropic displacement parameters (Å^2^)

**Table:**

	*x*	*y*	*z*	*U*_iso_*/*U*_eq_	
O1	0.32039 (6)	−0.14609 (12)	0.79180 (10)	0.0270 (3)	
O2	0.44401 (6)	0.09623 (12)	0.85186 (10)	0.0273 (3)	
N1	0.09135 (9)	0.06805 (17)	0.32350 (12)	0.0300 (4)	
C1	0.22426 (10)	0.16821 (19)	0.50087 (14)	0.0249 (4)	
C2	0.23245 (10)	0.01522 (19)	0.58506 (14)	0.0253 (5)	
C3	0.30568 (10)	−0.00345 (17)	0.70163 (14)	0.0230 (4)	
C4	0.37318 (9)	0.13126 (18)	0.73672 (14)	0.0238 (4)	
C5	0.36408 (10)	0.28352 (19)	0.65593 (14)	0.0267 (5)	
C6	0.28914 (10)	0.30120 (19)	0.53867 (14)	0.0268 (5)	
C7	0.14929 (10)	0.1882 (2)	0.37154 (14)	0.0271 (5)	
C8	0.02298 (10)	0.1056 (2)	0.19086 (15)	0.0328 (5)	
C9	0.03674 (10)	−0.01461 (19)	0.06882 (14)	0.0280 (5)	
C10	0.25804 (11)	−0.2911 (2)	0.75602 (16)	0.0335 (5)	
C11	0.50866 (10)	0.2352 (2)	0.89969 (16)	0.0334 (5)	
H2	0.186873	−0.076684	0.56133	0.0303*	
H5	0.40895	0.376514	0.680225	0.032*	
H6	0.282503	0.407674	0.483197	0.0322*	
H7	0.144082	0.298205	0.320967	0.0325*	
H8a	0.02928	0.225547	0.163206	0.0393*	
H8b	−0.039196	0.0907	0.208212	0.0393*	
H9a	0.098065	0.003565	0.048977	0.0337*	
H9b	0.034701	−0.13463	0.098931	0.0337*	
H10a	0.278325	−0.386148	0.821663	0.0402*	
H10b	0.195898	−0.256261	0.763371	0.0402*	
H10c	0.257961	−0.328557	0.658943	0.0402*	
H11a	0.55323	0.197841	0.983561	0.0401*	
H11b	0.540945	0.265307	0.82402	0.0401*	
H11c	0.475258	0.335902	0.923628	0.0401*	

### Atomic displacement parameters (Å^2^)

**Table:**

	*U*^11^	*U*^22^	*U*^33^	*U*^12^	*U*^13^	*U*^23^
O1	0.0291 (6)	0.0255 (5)	0.0232 (5)	−0.0036 (4)	−0.0026 (4)	0.0037 (4)
O2	0.0245 (5)	0.0302 (6)	0.0231 (5)	−0.0033 (4)	−0.0049 (4)	0.0017 (4)
N1	0.0301 (7)	0.0343 (7)	0.0218 (6)	0.0028 (6)	−0.0036 (5)	−0.0020 (5)
C1	0.0275 (8)	0.0271 (8)	0.0189 (7)	0.0039 (6)	0.0018 (6)	−0.0010 (6)
C2	0.0246 (7)	0.0285 (8)	0.0213 (7)	−0.0002 (6)	0.0014 (6)	−0.0043 (6)
C3	0.0264 (7)	0.0236 (8)	0.0182 (7)	0.0025 (6)	0.0024 (5)	0.0000 (6)
C4	0.0230 (7)	0.0293 (8)	0.0176 (7)	0.0017 (6)	0.0009 (6)	−0.0020 (6)
C5	0.0276 (8)	0.0278 (8)	0.0234 (7)	−0.0032 (6)	0.0021 (6)	−0.0005 (6)
C6	0.0319 (8)	0.0272 (8)	0.0208 (7)	0.0021 (6)	0.0037 (6)	0.0019 (6)
C7	0.0308 (8)	0.0284 (8)	0.0200 (7)	0.0057 (7)	0.0003 (6)	−0.0008 (6)
C8	0.0307 (8)	0.0365 (9)	0.0262 (8)	0.0043 (7)	−0.0059 (6)	−0.0004 (7)
C9	0.0252 (8)	0.0302 (8)	0.0258 (8)	−0.0001 (6)	−0.0019 (6)	0.0027 (6)
C10	0.0362 (9)	0.0277 (8)	0.0326 (8)	−0.0072 (7)	−0.0022 (7)	0.0042 (6)
C11	0.0283 (8)	0.0373 (9)	0.0299 (8)	−0.0088 (7)	−0.0052 (6)	0.0016 (7)

### Geometric parameters (Å, °)

**Table:**

O1—C3	1.3706 (16)	C6—H6	0.96
O1—C10	1.4272 (18)	C7—H7	0.96
O2—C4	1.3651 (14)	C8—C9	1.517 (2)
O2—C11	1.4287 (17)	C8—H8a	0.96
N1—C7	1.2657 (19)	C8—H8b	0.96
N1—C8	1.4620 (17)	C9—C9^i^	1.5236 (17)
C1—C2	1.403 (2)	C9—H9a	0.96
C1—C6	1.383 (2)	C9—H9b	0.96
C1—C7	1.4722 (17)	C10—H10a	0.96
C2—C3	1.3763 (17)	C10—H10b	0.96
C2—H2	0.96	C10—H10c	0.96
C3—C4	1.4139 (19)	C11—H11a	0.96
C4—C5	1.380 (2)	C11—H11b	0.96
C5—C6	1.3951 (18)	C11—H11c	0.96
C5—H5	0.96		
			
C3—O1—C10	117.08 (10)	N1—C8—C9	111.13 (12)
C4—O2—C11	116.78 (10)	N1—C8—H8a	109.4714
C7—N1—C8	117.04 (12)	N1—C8—H8b	109.4713
C2—C1—C6	119.20 (11)	C9—C8—H8a	109.4714
C2—C1—C7	121.32 (13)	C9—C8—H8b	109.471
C6—C1—C7	119.46 (13)	H8a—C8—H8b	107.7587
C1—C2—C3	120.15 (13)	C8—C9—C9^i^	112.43 (12)
C1—C2—H2	119.9235	C8—C9—H9a	109.4708
C3—C2—H2	119.9231	C8—C9—H9b	109.4709
O1—C3—C2	125.24 (12)	C9^i^—C9—H9a	109.4717
O1—C3—C4	114.61 (11)	C9^i^—C9—H9b	109.4713
C2—C3—C4	120.14 (12)	H9a—C9—H9b	106.344
O2—C4—C3	114.99 (11)	O1—C10—H10a	109.4718
O2—C4—C5	125.24 (12)	O1—C10—H10b	109.4712
C3—C4—C5	119.77 (11)	O1—C10—H10c	109.4714
C4—C5—C6	119.46 (13)	H10a—C10—H10b	109.471
C4—C5—H5	120.2676	H10a—C10—H10c	109.4712
C6—C5—H5	120.2684	H10b—C10—H10c	109.4707
C1—C6—C5	121.22 (13)	O2—C11—H11a	109.4712
C1—C6—H6	119.3878	O2—C11—H11b	109.4717
C5—C6—H6	119.3886	O2—C11—H11c	109.4713
N1—C7—C1	123.46 (13)	H11a—C11—H11b	109.4716
N1—C7—H7	118.272	H11a—C11—H11c	109.4708
C1—C7—H7	118.2721	H11b—C11—H11c	109.4708

Symmetry codes: (i) −*x*, −*y*, −*z*.

### Hydrogen-bond geometry (Å, °)

**Table:**

*D*—H···*A*	*D*—H	H···*A*	*D*···*A*	*D*—H···*A*
C11—H11A···O1^ii^	0.96	2.54	3.4945 (17)	171
C11—H11B···O1^iii^	0.96	2.58	3.4830 (17)	158

Symmetry codes: (ii) −*x*+1, −*y*, −*z*+2; (iii) −*x*+1, *y*+1/2, −*z*+3/2.
